# Time to recovery and its predictors among critically ill patients on mechanical ventilation from intensive care unit in Ethiopia: a retrospective follow up study

**DOI:** 10.1186/s12873-022-00689-3

**Published:** 2022-07-12

**Authors:** Lehulu Tilahun, Asressie Molla, Fanos Yeshanew Ayele, Aytenew Nega, Kirubel Dagnaw

**Affiliations:** 1 Department of Emergency and Ophthalmic Health, Wollo University, Dessie, Ethiopia; 2 School of Public Health, Department of Epidemiology and Biostatistics, Wollo University, Dessie, Ethiopia; 3 School of Public Health, Wollo University, Dessie, Ethiopia; 4 Desssie Comprehensive Specialized Hospital, Department of Intensive Care Unit, Dessie, Ethiopia; 5 Department of Comprehensive Health, Wollo University, Dessie, Ethiopia

**Keywords:** Artificial respiration, Intensive care unit, Mechanical ventilation, Predictor, Recovery, Time

## Abstract

**Introduction:**

For critically ill patients, mechanical ventilation is considered a pillar of respiratory life support. The mortality of victims in intensive care units is high in resource-constrained Sub-Saharan African countries. The recovery and prognosis of mechanically ventilated victims are unknown, according to evidence. The goal of the study was to see how long critically ill patients on mechanical ventilation survived.

**Methods:**

A retrospective follow-up study was conducted. A total of 376 study medical charts were reviewed. Data was collected through reviewing medical charts. Data was entered into Epi-data manager version 4.6.0.4 and analyzed through Stata version 16. Descriptive analysis was performed. Kaplan- Meier survival estimates and log rank tests were performed. Cox proportional hazard model was undertaken.

**Results:**

Median recovery time was 15 days (IQR: 6–30) with a total recovery rate of 4.49 per 100 person-days. In cox proportional hazard regression, diagnosis category {AHR: 1.690, 95% CI: (1.150- 2.485)}, oxygen saturation {AHR: 1.600, 95% CI: (1.157- 2.211)}, presence of comorbidities {AHR: 1.774, 95% CI: (1.250–2.519)}, Glasgow coma scale {AHR: 2.451, 95% CI: (1.483- 4.051)}, and use of tracheostomy {AHR: 0.276, 95% CI: (0.180–0.422)} were statistically significant predictors.

**Discussion:**

Based on the outcomes of this study, discussions with suggested possible reasons and its implications were provided.

**Conclusion and Recommendations:**

Duration and recovery rate of patients on mechanical ventilation is less than expected of world health organization standard. Diagnosis category, oxygen saturation, comorbidities, Glasgow coma scale and use of tracheostomy were statistically significant predictors. Mechanical ventilation durations should be adjusted for chronic comorbidities, trauma, and use of tracheostomy.

**Supplementary Information:**

The online version contains supplementary material available at 10.1186/s12873-022-00689-3.

## Introduction

The main objective of this paper was to study on time to recovery and its predictors among critically ill patients on mechanical ventilation from Intensive Care Unit (ICU) at Dessie Comprehensive Specialized Hospital, South Wollo Zone, Ethiopia.

Mechanical ventilation (MV) is defined as a life-sustaining and indispensable [1] mechanism intended to support or substitute normal breathing/lung function [2] when a patient is admitted to the ICU due to different reason like type I or type II Respiratory Failure (RF). In contrast to noninvasive ventilation, which primarily uses a barrier of a face mask, mechanical ventilation includes a breathing machine and an endotracheal tube (ETT) [3].

Globally, the number of patients requiring MV is highly increased, especially in older and comorbid patients. Due to this reason, intensive care medicine is a new specialty increasingly needed and which is supported with advanced technology in medical and scientific areas for the purpose of managing critically ill victims [4] who otherwise died [5]. That in turn creates health care planners to give emphasis to this type of medicine since patients consume extensive medical resources [4].

An invasive MV considered as pillar and cornerstone of respiratory life support among those in critically ill victims. The mortality rate and the economy of management of patients in MV remain high even though there is advancement in management of patients in MV [3].

Among those patients admitted from the ICU, around 35–50% require a respiratory support with MV. From those admitted to the ICU, an estimated 35–40% will die from the hospital. The recovery of patients that are managed by the use of MV depends on factors at the start of MV, during the management and at the time of complication [6].

It is assumed that the average median survival time from mechanical ventilation of patients admitted from an ICU is 11 days in pediatrics and 16 days in adult populations [1].

52% of patients admitted to the ICU for the purpose of mechanical ventilation have overall mortality. Almost all current persons might face an ICU environment during their life stay [7]. Greater than 50% of patients discharged from ICU, experience and ICU-related weakness and high linkage of death [8].The cost of admission for the purpose of mechanical ventilation is remarkably high[7, 9]. From all patients in a hospital, an ICU incurs an estimated 20% of medical expenses [10].

It is assumed that an extended period of admission at the ICU ends up with communicable disease, depression, and even mortality. For instance, around 80% of victims, admitted for an extended period end up with psychological disorders and traumatic brain injury [11].

In a similar way, in sub-Saharan Africa mortality rate of victims at an ICU is significant. Because of this, critically ill victims death rates with resource limited an ICUs frequently high[12]. In Ethiopia it is considered as mortality of patients admitted to an ICU is 40%[13]. Also from Ethiopian context, due to lack of resources (ICU beds, Mechanical Ventilators and trained staff), health personals have difficulties in making decisions [14].

The main predictors identified for survival of patients admitted for ICU were age, time of receiving mechanical ventilation, clients secondary to surgical operation, tracheostomy and hospital acquired disease [11], sex [15], trauma, poisoning [16], mechanical ventilator, mental status and length of ICU stay [17], increased monitoring[16]. Reports depict that, there is a lack of adequate data on determining issues in outcome of critically ill patients for survival in one or more weeks of MV in ICU [18].

## Materials and methods

The study was conducted at Dessie Comprehensive Specialized Hospital located in Amhara Regional State. The hospital is intended to serve 5 million populations and there are more than 118 patents admitted to the ICU for the purpose of mechanical ventilation per year. The study was conducted from January 1, 2016- December 31, 2020.

A retrospective follow-up study was conducted among critically ill patients admitted at an ICU for the purpose of mechanical ventilation at Dessie Comprehensive Specialized Hospital, South Wollo Zone, Ethiopia.

All adult critically ill patients admitted to the ICU for the purpose of mechanical ventilation from January 1, 2016- December 31, 2020, were included from the study. Pediatric age groups and those patient charts with incomplete baseline medical data specifically for variables not recorded like ICU admission date/MV initiation time, duration of mechanical ventilation, ICU discharge date, sociodemographics like (age & sex) and GCS were excluded from the study.

The final sample size was estimated through power and sample size determination for survival studies using Stata version 16 with stpower log rank through Schoenfeld method. The total sample size executed was 400 with 95% C.I, 80% power, with expected censoring rate (5%). Of 590 total ICU patients on mechanical ventilation during January 1, 2016- December 31, 2020, 400 medical charts were randomly selected using a simple random sampling technique. Then a total of 376 medical charts were actually included in the study. Twelve medical charts were incomplete. 7 charts were not available, and 5 were patients excluded from the study prior to commencing data collection.

A review of medical charts, was used as a data collection method. Actually, Data was collected during a data collection period of March 30- April 24, 2021. Eligible medical charts were selected when important variables were recorded. A research checklist taken from [1, 6, 16, 19–27] was used to collect data for the study. The main event of interest for this study was critically ill patients on mechanical ventilation discharged with recovery.

As per this study, *Time to recovery means* status of patient from time of admission to ICU for the purpose of mechanical ventilation to discharge with recovery (in days). The starting and ending time for the study shall be the time of ICU admission and the time of ICU release. As per the study area, ICU protocol only mandatory patients requiring MV admitted to ICU so that with a maximum of 30 min, patients get MV. *Event* is those of medical charts of critically ill patients labeled as discharged with recovery among those admitted for the purpose of mechanical ventilation at ICU. Censored are those of critically ill patients with death, not being discharged/ventilator dependent, or unknown outcome status (missed outcome during data collection).

To ensure study quality, a one-day training was given for five data collectors (BSC emergency nurses) to have a common understanding. In addition, the data collection period was supervised by the principal investigator and supervisor for appropriate data collection as per the time frame.

The collected data was coded and entered into Epi-data manager statistical software (version 4.6.0.4) and exported and analyzed using Stata version 16, and out puts were displayed using tables and charts. Summary statistics were performed by using frequencies, percentages and other descriptive measures. A Kaplan- Meier (KM) survival estimate was performed to plot the overall survival curve and to observe categorical predictors graphically. A log rank test was run to compare the difference in recovery among different predictors. A Schoenfeld global test and a graphical test of survival were undertaken to check for proportional hazard assumptions. Model goodness of fit was estimated Cox-Snell residual and Harrell’s C concordance statistic test.

A cox proportional hazard model, through a bi-variable and multi-variable analysis was undertaken to identify the effect of predictors on time of recovery on mechanical ventilation. So that P values, adjusted hazard ratio (AHR), and 95% confidence intervals were calculated to estimate simultaneous effects of covariates on events of interest.

## Results

### Description of the study participants

**Fig. 1:**
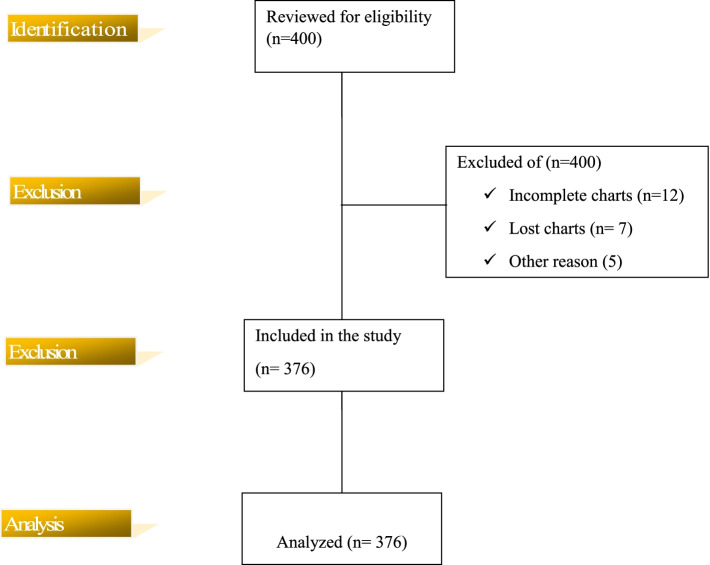
STROBE flow chart of critically ill patients on mechanical ventilation from intensive care unit at Dessie Comprehensive specialized, Ethiopia. (*n* = 376). actual subjects from identification till analysis stage Yellow shaded area on left: indicates stages of flow of chart. Unshaded area on right side: shows actual subjects from identification till analysis stage

In the study of critically ill patients, a total of 590 patients were admitted to an ICU for the purpose of mechanical ventilation during the study period of January 1, 2016- December 31, 2020. Of them, 400 study subjects were randomly sampled to conduct the study and 376 medical charts were reviewed ( \* MERGEFORMAT Fig. 1).

### Demographic and clinical characteristics

The mean age for the study participants was 40.73 ± 16.27 years (range, 18–90 years). Above half 217(57.7%) were males. In relation to oxygen saturation, the majority of 252(67.0%) of them recorded as ≤ 90%. Most 172(45.7%) subjects measured as 9–12 Glasgow coma scale, followed by ≤ 8 GCS counted as 156(41.5%). On the other hand, this document review depicted that, the majority 207 (55.1%) of participants were due to type II respiratory failure (RF) that contributed to initiation of mechanical ventilation. In a similar manner, almost half 170(45.2%) study subjects were due to trauma & burn diagnosis category followed by abdominal, respiratory, and cardiac diagnosis categories, which counted as 40(10.6%), 39(10.4%) and 37(9.8%) respectively.

The emergency department (ED) was the main source of admission to the ICU which accounted for 229(60.9%). Among respondents, 144(38.3%) had comorbidities. In relation to place of intubation, a majority 331(88.0%) were reviewed as receiving mechanical ventilation with in the ICU. In addition, only 94(25%) of the study participants received a tracheostomy during their stay at an ICU ( \* MERGEFORMAT Table 1).

**Table 1:** Demographic and clinical characteristics of study participants from Intensive care unit at Dessie Comprehensive specialized, Ethiopia, 2021 (*n* = 376)

Variables	Category	Frequency(n)	Percent (%)
Age	All	40.73 ± 16.27	
Sex	Male	217	57.7
	Female	159	42.3
Admission V/S			
Respiratory rate	Less than 12	10	2.7
	12–20	70	18.6
	Greater than 20	296	78.7
Pulse rate	< 60	21	5.6
	60–100	138	36.7
	> 100	217	57.7
Oxygen Saturation	< 90	225	59.8
	≥ 90	151	40.2
Glasgow Coma Scale	≤ 8	156	41.5
	9–12	172	45.7
	13–15	48	12.8
Causes of Initiation of Mechanical Ventilation	Respiratory type I	146	38.8
	Respiratory type II	206	54.8
	Coma	23	6.1
	Other causes(Specify) ^*^	1	0.3
Source Of Admission	Emergency Department	229	60.9
	Medical ward	49	13
	Surgical ward	48	12.8
	Gynecology/Obstetrics ward	19	5.1
	Operation Room	30	8
	Other (Specify) ^**^	1	0.3
Diagnosis Category at time of Receiving Mechanical ventilation	Trauma, Burn	170	45.2
	Poisoning	13	3.5
	Respiratory	39	10.4
	Cardiac	37	9.8
	Abdomen	40	10.6
	Malignancy	8	2.1
	Endocrine	12	3.2
	Nervous system	27	7.2
	Obstetric	26	6.9
	Other(Specify) ^***^	4	1.1
Comorbidity	Yes	144	38.3
	No	232	61.7
Place of Intubation	In Intensive care unit	331	88
	Out Intensive care unit	45	12
Use of Tracheostomy	Yes	94	25.0
	No	282	75.0
	Total	376	100.0

^*^ DVT

^**^ cancer unit

^***^ Septic shock, Gangrenous, Empyema

### Time to recovery of critically ill patients on mechanical ventilation

**Fig. 2:**
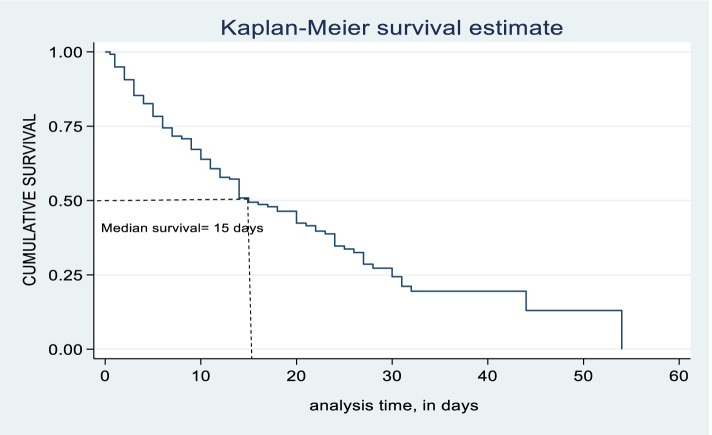
Kaplan–Meier survival estimate of recovery time of critically ill patients on mechanical ventilation from intensive care unit at Dessie Comprehensive specialized, Ethiopia, 2021. (n = 376). Horizontal axis (X): shows time of analysis in (days). Vertical axis (Y): indicates cumulative survival. Middle line (down ward) Survival function

Of 376 total observations, 159 (42.29%) with 95%C.I of (37.4%-47.4%) critically ill patients have recovered. Among censored observations, 127(33.78%) died. 9(2.39%) were ventilator-dependent. This research resulted in a median recovery time for critically ill patients on mechanical ventilation was 15 days with an interquartile range (IQR) of (6–30 days). So the total person-day risk estimated was 3543 person-days. The total recovery rate of critically ill patients on mechanical ventilation was 4.49 per 100 person- days (95%CI: 3.84–5.24). Similarly, as per the Kaplan–Meier survival cure for 60-day of critically ill patients on mechanical ventilation, survival plot decreases swiftly as time elapses. So that there is a decrease in survival of critically ill patients on mechanical ventilation as number of days increases. In 60-day critically ill patients on mechanical ventilation analysis, the highest recovery of critically ill patients on mechanical ventilation was observed at fifth day with survival of 78.3% (95% C.I: 73.4–82.5) and tenth day with recovery of 63.9% (95% C.I: 57.8–69.3) days respectively. So, the recovery rate of critically ill victims on mechanical ventilation was 8 per 100 person-days and 3.7 per 100 person-days at 5th and 10th days respectively ( \* MERGEFORMAT Table 2, \* MERGEFORMAT Fig. 2 & \* MERGEFORMAT Table 3).

**Table 2:** Kaplan–Meier estimate of survivor function by time variable of study participants from Intensive care unit at Dessie Comprehensive specialized, Ethiopia, 2021 (*n* = 376)

Time (days)	Beg. total	Fail	Survivor Function	95%C.I
0.5	376	3	0.9920	0.976–0.997
5	232	71	0.7832	0.734–0.825
10	141	34	0.6385	0.578–0.693
15	71	24	0.4942	0.425–0.560
20	58	9	0.4239	0.351–0.495
25	34	9	0.3368	0.261–0.414
30	19	7	0.2438	0.168–0.328
35	9	3	0.1951	0.121–0.282
40	5	0	0.1951	0.121–0.282
45	3	1	0.1300	0.042–0.270
50	2	0	0.1300	0.048–0.269

**Table 3:** Median time recovery and comparison of survival among study participants from Intensive care unit on mechanical ventilation at Dessie Comprehensive specialized, Ethiopia, 2021. (*n* = 376)

Variable	Category	Median recovery time (Point estimate)	95% CI	Log rank test, *p*-value
All	IQR = 6–30	15	14–20	0.128
Sex	Male	20	14–24	
	Female	14	11–20	0.188
Type of respiratory failure	Type I	14	10–18	
	Type II	20	14–25	0.797
Source of Admission	In ED	17	14–21	
	Out ED	14	11–24	0.001
Diagnosis category at time of receiving MV	Trauma	22	16–28	
	Non-trauma	12	9–15	0.927
Place of Intubation	In ICU	15	14–20	
	Out ICU	15	9–21	0.143
Respiratory rate	≤ 20	14	9–20	
	> 20	18	14–23	0.610
Pulse rate	< 60	18	5–25	
	60–100	14	10–20	
	> 100	20	13–24	
Oxygen Saturation	< 90	20	15–25	0.014
	≥ 90	13	9–15	0.008
Comorbidities	Yes	24	15–31	
	No	13	11–17	0.012
Glasgow Coma Scale	≤ 8	22	10–20	
	9–12	14	9–15	
	13–15	14	11–17	
Use of tracheostomy	Yes	24	20–28	< 0.001
	No	11	9–12	

### Test for equality of survival function

A Log-Rank test was run to see the difference in survival time between different predictor variable categories. As a result variables like diagnosis category at time of receiving, mechanical ventilation, oxygen saturation, presence of comorbidities, Glasgow coma scale, and use of tracheostomy had significant survival differences. On the contrary, survival time among sex, type of respiratory failure, source of admission, place of endotracheal intubation, admission-pulse rate and admission respiratory rate did not show differences at a *P*-value of < 0.05 ( \* MERGEFORMAT Table 3).

**Fig. 3:**
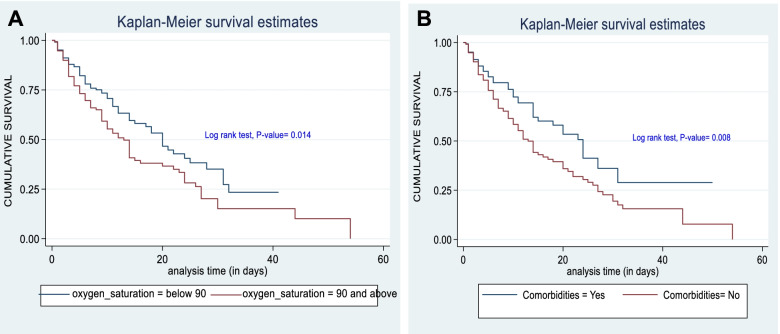
A. Kaplan–Meier survival estimate by oxygen saturation of study participants from intensive care unit at Dessie Comprehensive specialized, Ethiopia, 2021. (*n* = 376). B. Kaplan–Meier survival estimate by comorbidity of study participants from intensive care unit at Dessie Comprehensive specialized, Ethiopia, 2021. (*n* = 376). Horizontal axis (X): shows time of analysis in (days). Vertical axis (Y): indicates cumulative survival. Middle-blue line (down ward) Survival of oxygen saturation >  = 90%. Middle-red line (down ward) Survival of oxygen saturation < 90%. Middle-blue line (down ward): Survival of those having comorbidities. Middle-red line (down ward) Survival of those not having comorbidities

Graphically, for instance, there is a significant survival difference between with those of ≤ 90% oxygen saturation and those study participants having oxygen saturation of greater than 90% in addition between those having comorbidities and those not having comorbidities due to their gap of two curves in each graph and log rank test p-value of two curves per each graph rejects assumption of equality ( \* MERGEFORMAT Fig. 3A & Fig. 3B).

### Predictors of time to recovery of critically ill patients on mechanical ventilation

The overall Schoenfeld global test graphical test did not violate the proportional hazard assumption at (X2 = 5.88; *P*-value = 0.8250) ( \* MERGEFORMAT Table 4). Multi-collinearity was not suspected between predictor variables as per the Pearson correlation coefficient and variance inflation factor. So, the correlation matrix revealed with absolute Pearson Correlation coefficient was less than 0.47 and also the mean variance inflation factor (VIF) of predictors was ≤ 1.11 (min: 1.02; max: 1.36).

**Table 4:** Cox proportional hazard regression of predictors on recovery of critically ill patients on mechanical ventilation at Dessie Comprehensive specialized, Ethiopia, 2021. (*n* = 376)

Predictors	Category	Recovery		CHR(95% CI)	AHR (95% CI)	*P*-value
		Event	Censored			
Sex	Male	80	137	0.783(0.573- 1.070)	0.758(0 .547- 1.050)	0.095
	Female	79	80	1	1	
Age	40.73 ± 16.27	40.73 ± 16.27		0.992(0.982- 1.003)	0.992(0.981–1.00)	0.106
Type of respiratory failure	Type I	66	81	0.807(0.904- 1.697)	0.914( 0.631–1.325)	0.635
	Type II	93	136	1	1	
Diagnosis category at time of receiving MV	Trauma	55	115	1	1	
	Non-trauma	104	102	1.722(1.2411–2.391)	1.690( 1.150- 2.485)	0.008*
Respiratory rate	≤ 20	43	37	1.301(0.916- 1.848)	1.417(0 .983- 2.044)	0.062
	> 20	116	180	1	1	
Oxygen Saturation	< 90	75	150	1	1	
	≥ 90	84	67	1.481(1.083- 2.027)	1.600(1.157- 2.211)	0.004 ^*^
Comorbidities	Yes	47	97	1	1	
	No	112	120	1.578(1.121- 2.221)	1.774(1.250- 2.519)	0.001 ^*^
Glasgow Coma Scale	≤ 8	45	111	1	1	
	9–12	86	86	1.584(1.103- 2.274)	1.993( 1.358- 2.924)	< 0.001 ^*^
	13–15	26	20	1.865(1.162- 2.992)	2.451( 1.483- 4.051)	< 0.001 ^*^
Use of tracheostomy	Yes	51	43	0 .394(0.271- 0.572)	0.276(0.180–0.422)	< 0.001 ^*^
	No	108	174	1	1	

^*^ Statistically Significant at *P* < 0.05

**Fig. 4:**
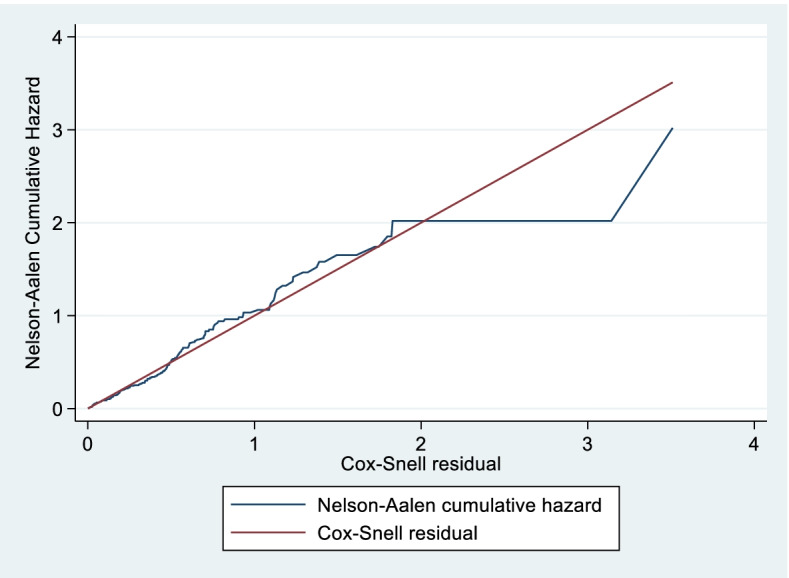
Cumulative hazard of Cox-Snell residuals of study participants from intensive care unit at Dessie Comprehensive specialized, Ethiopia, 2021. (*n* = 376). Horizontal axis (X): shows Cox Snell residual. Vertical axis (Y): indicates Nelson Aalen cumulative hazard. Middle-blue line (Jagged) indicates Nelson Aalen cumulative hazard. Middle-red line (45o) Cox Snell residual

The model fitness was estimated through Cox-Snell residual (indicating a fitted model about a straight 45o line) and Harrell’s C concordance statistic (resulted in a Harrell’s C value of 70.56) ( \* MERGEFORMAT Fig. 4). Predictors that ended up with P-value of less than 0.2 in bi-variables were further entered into multi-variable analysis. In cox proportional hazard regression, predictors were run by using an Efron method for handling ties of survival time. So that in multi-variable analysis, predictors such as diagnosis category at time of receiving mechanical ventilation, arterial oxygen saturation, presence of comorbidities, Glasgow coma scale, and use of tracheostomy were statistically significant at *P*-value of 0.05 level of significance with recovery of critically ill patients on mechanical ventilation. But Sex, age, type of respiratory failure, admission-respiratory rate and admission pulse rate were not statistically significant predictors of recovery of critically ill patients on mechanical ventilation.

At any particular time, non-traumatic patients had a 69.0% faster rate of recovery proportionally to traumatic patients on mechanical ventilation {AHR: 1.690; 95% CI: (1.150- 2.485); *P*-value: 0.008}. Oxygen saturation as an independent predictor, those patients having 90%, and above oxygen saturation had a 60% greater recovery rate than its counterpart {AHR: 1.600; 95% CI: (1.157- 2.211); *P*-value: 0.004}. The recovery time of critically ill patients with free of comorbidities was 77.4% greater as proportionally to those having comorbidities {AHR: 1.774; 95% CI: (1.250- 2.519); *P*-value: 0.001}. Glasgow comma Scale (GCS) as one predictor variable, patients with GCS scales of 9–12 had a 99.3% faster rate of recovery than patients with GCS scales of 8 or below {AHR: 1.993; 95% CI: (1.358- 2.924); *P*-value: < 0.001}. Similarly, at any time patients with GCS scale of 13–15 had two times faster recovery than patients with GCS of 8 or below {AHR: 2.451; 95% CI: (1.483- 4.051); *P*-value: < 0.001}. On the other hand, critically ill patients on mechanical ventilation receiving a tracheostomy had a 72.4% lower rate of recovery than its counter parts {AHR: 0.276; 95% CI: (0.180–0.422); *P*-value: < 0.001} ( \* MERGEFORMAT Table 5).

**Table 5:** Test of proportional hazard assumption with rank detail of data from critically ill patients on mechanical ventilation at Dessie Comprehensive specialized, Ethiopia, 2021. (*n* = 376)

S/N	Predictors	rho	X^2^	df	P-value
1	Sex: Ref: Female	0.03343	0.20	1	0.659
2	Age	-0.05451	0.52	1	0.470
3	Respiratory rate	-0.06111	0.63	1	0.426
4	Pulse rate	0.07885	0.99	1	0.320
5	Oxygen saturation: Ref: < 90	-0.00230	0.00	1	0.976
6	Comorbidities: Ref: Yes	0.03525	0.08	1	0.660
7	Glasgow Coma Scale: Ref: ≤ 8	0.03841	0.25	1	0.620
8	Diagnosis category: Ref: Trauma	-0.06052	0.66	1	0.418
9	Use of tracheostomy: Ref: No	-0.09239	1.30	1	0.254
10	Type of respiratory failure: Ref: Type II	-0.03971	0.28	1	0.596
	Global Test		5.88	10	0.825

## Discussion

This facility based retrospective follow-up study was mainly focused on time to recovery and its predictors among critically ill patients on mechanical ventilation. The study revealed that the median recovery time of critically ill patients on mechanical ventilation was 15 days with an interquartile range (IQR) of (6–30 days). This result is supported by findings from Saudi Arabia, India, Germany, Brazil, Nigeria, and Ethiopia [1, 3, 25, 26, 28, 29]. However, the finding is lower than studies from US & UK ICUs [30], Kenya [31], Nigeria [32], and University of Jimma, Ethiopia [33]. This might be because of difference in level of ICU organization and ICU supplies as well as it might be due to presence of long term acute care facilities and palliative care/hospice facilities in US & UK ICUs. This implies the need for refining quality ICU organization and related conditions in the study area. Therefore, it is recommended that the Federal Ministry of Health (FMOH) in collaboration with health facility and other supportive organizations should give its time to invest on ways to enhance ICU organization and essential ICU supplies in order for improving recovery rate of critically ill patients on mechanical ventilation.

This paper found that, in cox proportional hazard regression, predictors such as diagnosis category at time of receiving mechanical ventilation, oxygen saturation, presence of comorbidities, Glasgow coma scale, and use of tracheostomy were statistically significant.

At any particular time, non-traumatic critically ill patients had a 69.0% faster rate of recovery proportional to traumatic patients on mechanical ventilation at ICU {AHR: 1.690; 95% CI: (1.150- 2.485)}. This is supported by research results conducted in Sub-Saharan Africa [12]. The possible justification for this probably would be because of severe, accidental violence and road traffic injuries in the study area. In addition, it might be due to most of the time head involvement in these types of severe accidental injuries that end up with severe traumatic injuries (TBI). This implies that road traffic accidents, violence and other forms of injury look prevalent in the study area.

The recovery time of critically ill patients with free of comorbidities faced a 77.4% greater recovery as proportionally to those having comorbidities {AHR: 1.774; 95% CI: (1.250- 2.519)}. This study finding was supported by findings from Pakistan, Germany, Brazil, and Ethiopian studies [3, 4, 11, 25, 34].

At any time, those patients having 90% and above oxygen saturation had a 60% greater recovery than its counterparts {AHR: 1.600; 95% CI: (1.157- 2.211)}. Similarly, at any particular time, patients with GCS scale of 9–12 had 99.3% faster recovery rate relative to patients with GCS scale of 8 or below {AHR: 1.993; 95% CI: (1.358- 2.924)} and critically ill patients with GCS scale of 13–15 had two times more recovery time than patients with GCS of 8 or below {AHR: 2.451; 95% CI: (1.483- 4.051)}. These findings were supported by research output from Pakistan, the southern part of Ethiopia [4, 25].

On the other hand, critically ill patients on mechanical ventilation receiving a tracheostomy had a 72.3% lower rate of recovery than its counter parts {AHR: 0.277; 95% CI: (0.181–0.423)}. This research finding is comparable to finding in Sub Saharan Africa [12]. But it has a discrepancy with findings from tertiary hospitals of Nigeria [29] in which those gaining tracheostomy recovered better. This might be due to a difference in time of initiation of tracheostomy after intubation for mechanical ventilation.

This study also depicted that 42.29% critically ill patients were recovered with the overall recovery rate of critically ill patients on mechanical ventilation as 4.49 per 100 person- days. This finding is similar to that of studies from different areas of study. For instance in Southern Brazil Nigeria, Kenya, Jimma University Specialized Hospital, and also from a systematic review of 39 countries [3, 29, 31, 33, 35]. But, the finding was lower from other different parts of the world. For instance, finding from Saudi Arabia, Germany, India, Uganda, Egypt, South Western Kenya, Gondar Hospital, and Southern part of Ethiopia recovery [1, 12, 16, 17, 25–27, 36]. The possible explanation for ending up with lower finding could possibly be due to differences in the level of quality of ICU set-up and availability of intensive care equipment and due to the presence of long term acute care facilities and palliative care/hospice facilities in some countries. Even, for instance, some are studied from specialized weaning ICU centers. Plus, studies from Saudi Arabia and Germany mainly focused on tertiary care centers and specialized weaning ICU centers and also study from Kenya; it includes pediatric population unlike this study area. This indicates the importance of upgrading ICU, medical equipment, supplies and staffing. The hospital is better able to enhance its level of ICU quality, its staffing and ICU equipment. ICU health staff should give attention, frequent monitoring and expand their knowledge on handling of critical ill victims, those on mechanical ventilation.

The paper also demonstrated main causes of admission to the ICU for the purpose of mechanical ventilation were traumatic injuries (45.2%), abdominal (10.6%), respiratory (10.4%), and cardiac (9.8%) causes. This study finding is almost similar to studies conducted in Sub Saharan Africa, Nigeria, Uganda, South Western Kenya and Jimma University Specialized Hospital [12, 16, 32, 33] in which traumatic injuries were number one causes. However, finding from Sothern Brazil, India & Southern Ethiopia [3, 25, 37] ended up with malignancy, sepsis and cardiovascular & respiratory causes respectively were mainly explained reasons for admission to ICU for mechanical ventilation. The possible justification for trauma causes that made study participants visit an ICU for the purpose of mechanical ventilation was because of major occurrence of traumatic injuries and violence in the developing world. This signifies that road traffic accidents, violence and other trauma are the major events which adversely affected the lives of the population in the study area. It is advised that transport office should take serious measures be taken in order for preventing and controlling occurrence of traumatic injuries because almost half of critically ill victims on mechanical ventilation as per this research were accidental injuries and also it’s statistically significant.

Finally, since as per the knowledge of the researcher, there are no enough studies conducted on time to recovery among critically ill victims on mechanical ventilation and its predictors in Ethiopian context, so it’s advised for interested researchers to invest their time on this issue mainly by using prospective research approaches.

### Limitations of the study

This study was not without limitations; for instance, it’s using of time of admission, and discharge of critically ill victims, those on mechanical ventilation as time of observation interval due to lack of adequate record keeping. This was done because, as per the study area, only mandatory critically ill patients needing mechanical ventilation were admitted to ICU.

In addition, ventilator modes, and blood chemistry tests were not studied due to issues in record keeping, so that the effect of these conditions on recovery of the studied population were not determined.

## Conclusion

In conclusion, the study found that below half of the observations were recovered. The median recovery time and recovery rate of study participants were below expected.

In cox proportional hazard regression, predictors such as diagnosis category at time of receiving mechanical ventilation, arterial oxygen saturation, presence of comorbidities, Glasgow coma scale, and use of tracheostomy were statistically associated with recovery.

The major reason for visiting an ICU for the purpose of mechanical ventilation were traumatic injuries, followed by abdominal, respiratory and cardiac problems.

## Publisher’s Note 

Springer Nature remains neutral with regard to jurisdictional claims in published maps and institutional affiliations. 

## Supplementary Information

**Additional file 1:** 

**Additional file 2:** 

## Data Availability

All data generated or analyzed during this study are included in this published article [and its supplementary information files].

## Abbreviations

ED: Emergency department
ETT: Endotracheal Tube
FMOH: Federal Ministry of Health
GCS: Glasgow Coma Scale
ICU: Intensive Care Unit
KM: Kaplan- Meier
MV: Mechanical ventilation
PHA: Proportional Hazard Assumption
RF: Respiratory Failure
STROBE: Strengthening the Reporting of Observational Studies in Epidemiology
VIF: Variance Inflation Factor
