# Supplementary material for: Time to recovery and its predictors among critically ill patients on mechanical ventilation from intensive care unit in Ethiopia: a retrospective follow up study
Source: BMC Emerg Med. 2022 Jul 12;22:125. doi: 10.1186/s12873-022-00689-3 (PMC9277794; doi:10.1186/s12873-022-00689-3)
Supplement: Supplementary file 2 — Additional file 2: [file 12873_2022_689_MOESM2_ESM.docx]

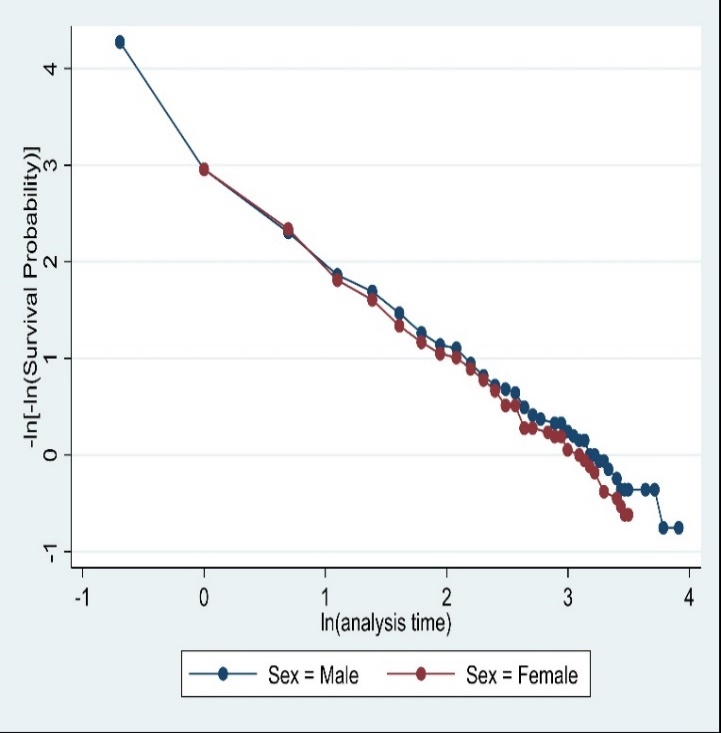

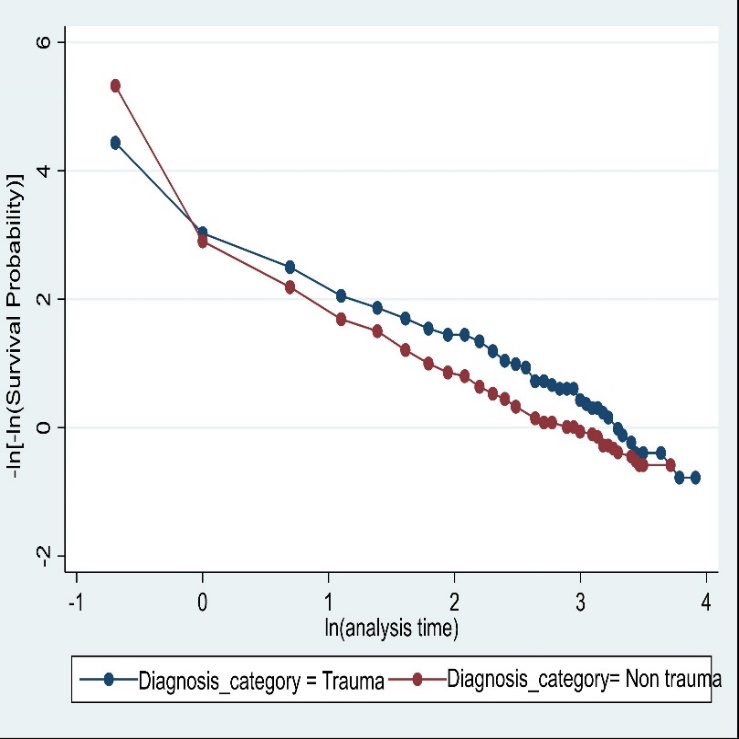


Figure 1. Test of proportional hazard assumption graphically by using sex and diagnosis category of critically ill patients on mechanical ventilation at Dessie Comprehensive specialized, Ethiopia, 2021. (n= 376).


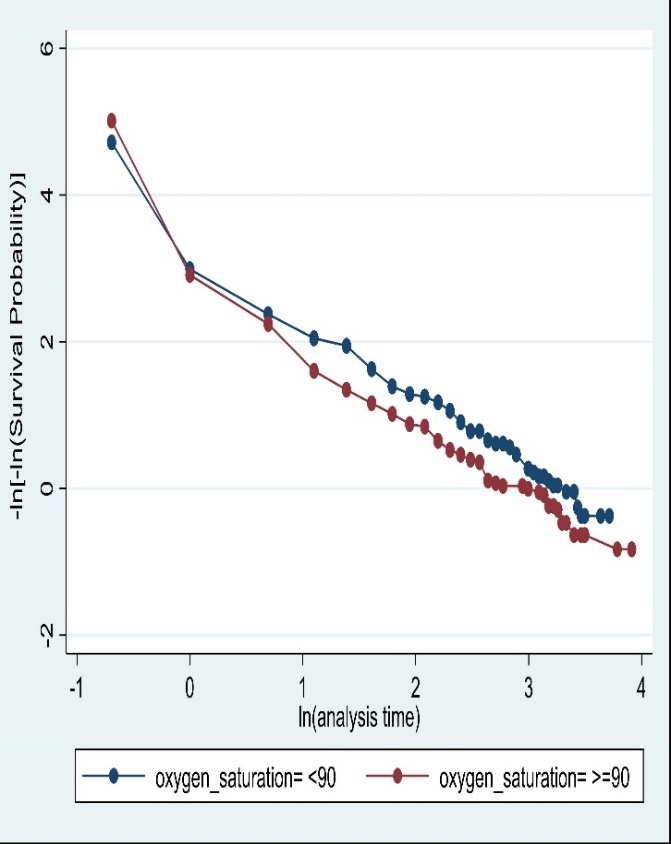

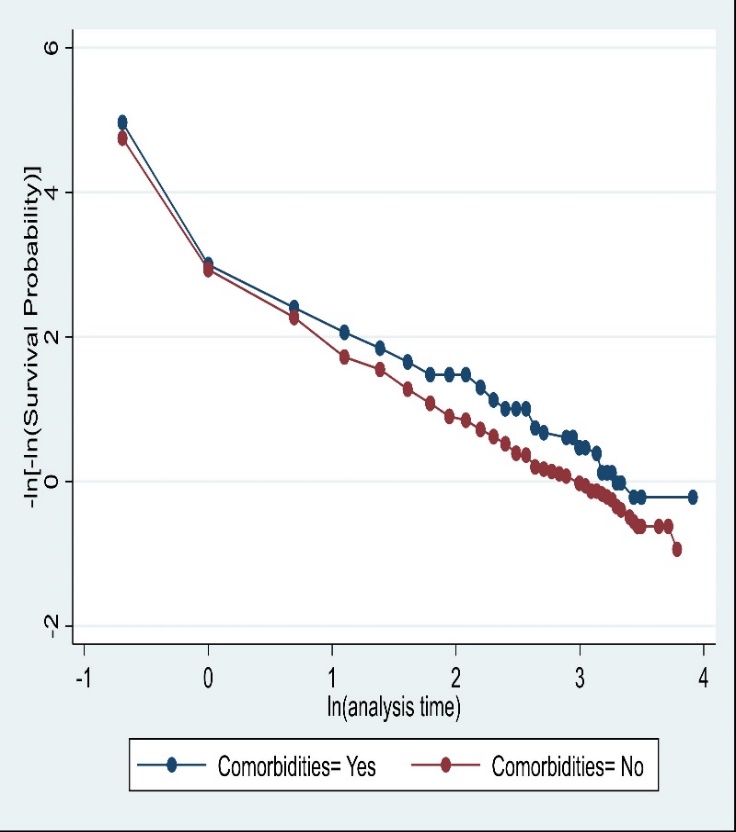


Figure 2. Test of proportional hazard assumption graphically by using oxygen saturation and comorbidities of critically ill patients on mechanical ventilation at Dessie Comprehensive specialized, Ethiopia, 2021. (n= 376).
